# Simultaneous Papillary Carcinoma in Thyroglossal Duct Cyst and Thyroid

**DOI:** 10.1155/2017/8541078

**Published:** 2017-02-08

**Authors:** Gustavo Cancela e Penna, Henrique Gomes Mendes, Adele O. Kraft, Cynthia Koeppel Berenstein, Bernardo Fonseca, Wagner José Martorina, Andreise Laurian N. R. de Souza, Gustavo Meyer de Moraes, Kamilla Maria Araújo Brandão Rajão, Bárbara Érika Caldeira Araújo Sousa

**Affiliations:** ^1^Federal University of Minas Gerais (UFMG), Belo Horizonte, MG, Brazil; ^2^Federal University of Rio de Janeiro, Rio de Janeiro, RJ, Brazil; ^3^Division of Endocrinology, Hospital Mater Dei, Belo Horizonte, MG, Brazil; ^4^Department of Pathology, Virginia Commonwealth University, Richmond, VA, USA; ^5^Division of Pathology, Instituto Roberto Alvarenga, Belo Horizonte, MG, Brazil; ^6^Division of Radiology, Spectra Institute, Belo Horizonte, MG, Brazil; ^7^Division of Endocrinology, Hospital Biocor, Belo Horizonte, MG, Brazil; ^8^Division of Endocrinology, Hospital da Baleia, Belo Horizonte, MG, Brazil; ^9^Division of Head and Neck Surgery, Hospital das Clinicas, UFMG, Belo Horizonte, MG, Brazil; ^10^Division of Endocrinology, Hospital das Clinicas, UFMG, Belo Horizonte, MG, Brazil; ^11^Division of Endocrinology, Hospital Mario Pena, Belo Horizonte, MG, Brazil

## Abstract

Thyroglossal duct cyst (TDC) is a cystic expansion of a remnant of the thyroglossal duct tract. Carcinomas in the TDC are extremely rare and are usually an incidental finding after the Sistrunk procedure. In this report, an unusual case of a 36-year-old woman with concurrent papillary thyroid carcinoma arising in the TDC and on the thyroid gland is presented, followed by a discussion of the controversies surrounding the possible origins of a papillary carcinoma in the TDC, as well as the current management options.

## 1. Background

Thyroglossal duct cyst (TDC) is the most common congenital, benign, midline neck mass, accounting for 7% of midline neck swellings in adults [1]. A TDC arises as a cystic expansion of a remnant of the thyroglossal duct tract and is the most frequent congenital anomaly of the neck [2].

Associated carcinoma is extremely rare, occurring in about 1% of TDC cases [3], with fewer than 300 cases reported since the first description by Brentano in 1911 [4]. The clinical presentation of a TDC carcinoma (TDCCa) is often asymptomatic and very similar to its benign counterpart. Thus, it is difficult to identify the TDCCa on clinical examination, as well as on ultrasound, scintigraphy, or even at fine needle aspiration biopsy (FNAB) and the diagnosis of the malignancy is generally incidental after surgery [5].

The majority of carcinomas are small (0.2 cm to 1.5 cm) and confined to the cyst, papillary thyroid being the most common histological type [6, 7]. The average patients' age is 40 years old and it is more frequent in females [8]. The finding of a carcinoma in a TDC after adequate excision of the cyst, usually by means of the Sistrunk procedure (SP), is a surprise for both the patient and the physician [5].

It is still debated whether TDCCa originates from the thyroid gland, from the TDC itself (de novo theory) or from both [9–12]. Although the differentiation may be difficult, this distinction can play a crucial role in treatment decisions regarding the inclusion of thyroidectomy as part of the treatment strategy versus TDC total resection exclusively [13].

## 2. Case Presentation

A 36-year-old female patient presented with a slow-growing, painless, midline neck mass. She reported no previous radiation exposure and no signs or symptoms of thyroid abnormalities, hoarseness, breathing difficulty, or dysphagia. Her medical records were reviewed and her medical history was otherwise unremarkable. Physical examination revealed a smooth, well-circumscribed mass along the midline of the neck, overlying the thyrohyoid membrane, mobile with deglutition and protrusion of the tongue. There were no palpable lymph nodes.

## 3. Investigation

**Figure 1:**
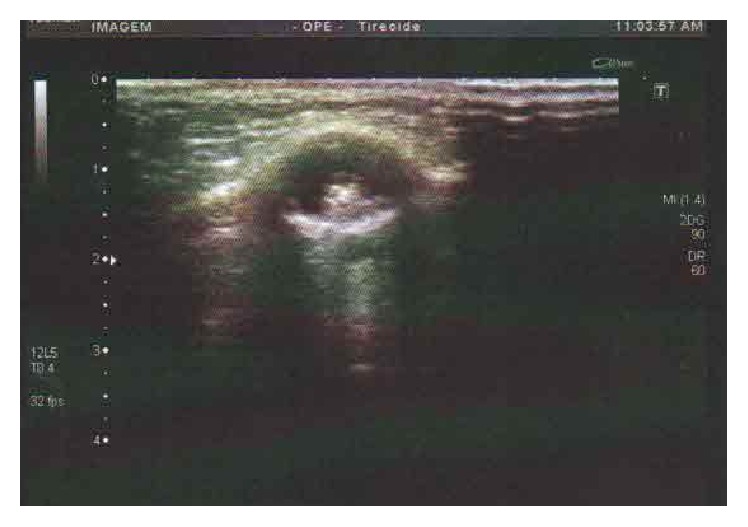
Sonography shows an image suggestive of thyroglossal duct cyst containing debris and an eccentric small hyperechoic solid area.

Thyroid function tests were normal. The neck ultrasound revealed a pattern suggestive of a thyroglossal duct cyst: a single, midline, suprahyoid cyst containing debris and an eccentric small hyperechoic solid area. This structure measured 1.6 cm^3^ with absence of flow on Doppler-sonography (Figure 1). The midline neck location and the close relationship between the lesion and the hyoid bone were considered the key to the differential diagnosis of the TDC, which includes branchial cleft cysts and lymph nodes [14]. Because the frequency of thyroglossal cyst carcinoma is very low, in a large percentage of cases the clinicians seldom consider an oncologic diagnosis and therefore do not perform a preoperative fine needle aspiration biopsy, albeit its low sensitivity [15].

After a fully informed written consent, complete excision of thyroglossal duct with central thyroidectomy was performed (standard Sistrunk procedure). The surgical aspect was of that of a thyroglossal duct cyst. The lesion was dissected up to the hyoid bone and then to the base of the tongue. No abnormal findings were observed intraoperatively and there were no intercurrences or complications.

**Figure 2:**
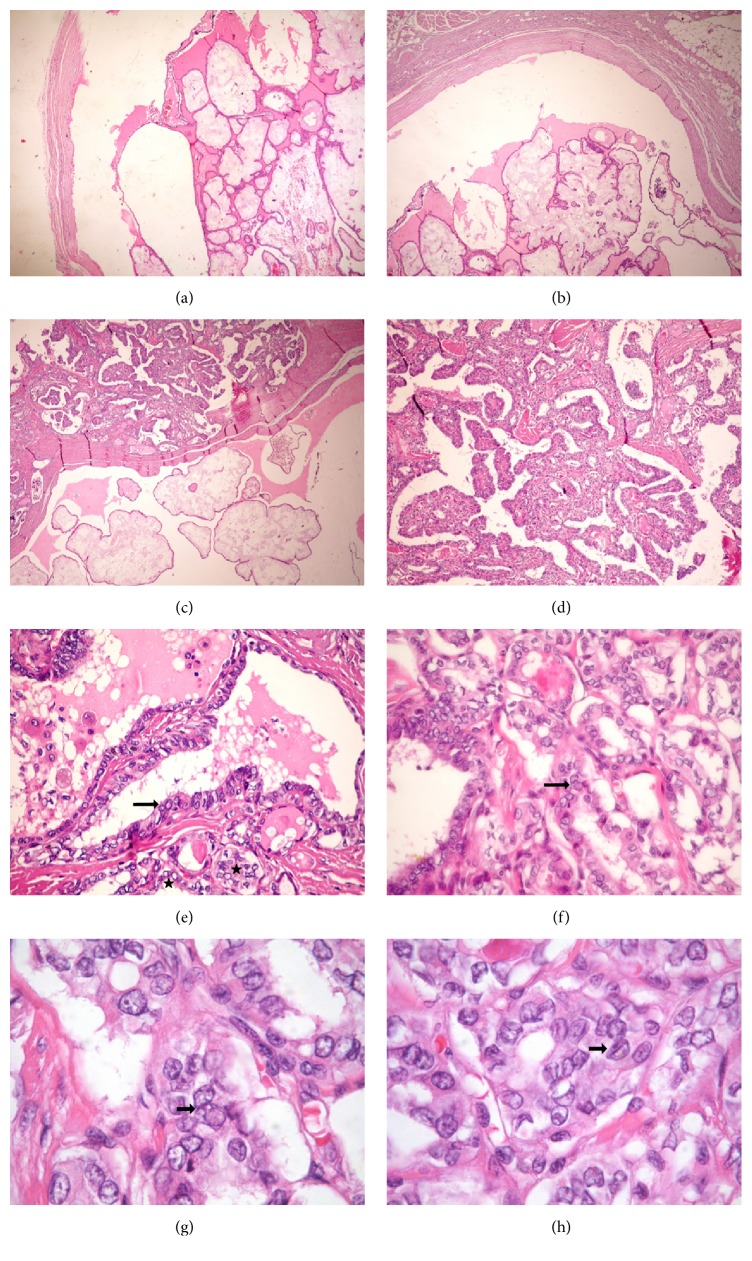
Thyroglossal duct cyst carcinoma. (a) and (b) Low power view of the cyst containing the papillary carcinoma (HE, 40x). (c) The papillae were sometimes edematous (HE, 40x). (d) The tumor had papillae, but also some follicles (HE, 100x). (e) Nuclei with pseudoinclusions (arrow) and ground glass appearance (stars) (HE, 400x). (f) Nuclear pseudoinclusion (HE, 400x). (g) Nuclear pseudoinclusion (arrow) (HE, 1000x). (h) Nuclear groove (arrow) (HE, 1000x).

Due to the characteristics observed on the ultrasound and the location of the lesion, it was assumed that it was a thyroglossal cyst. Gross examination showed a 3.0 cm cyst, filled with a gelatinous green material, with a bone fragment attached to it. The histopathological report revealed a cystic lesion and a tumor characterized by the proliferation of columnar cells in a single layer, mostly arranged in papillae, but also in follicles, supported by a richly vascularized connective tissue. The cells had ovoid, ground glass nuclei (“Orphan Annie” eye), sometimes with grooves and pseudoinclusions (Figures 2(a)–2(h)). The tumor measured 1.4 centimeter. There was minimal infiltration of the adjacent fibroadipose tissue. This histology was compatible with papillary carcinoma in the TDC and it was staged as pT3c N0 M0 [16].

The case was then presented at a multidisciplinary meeting, when all the clinical and radiological data were reviewed.

## 4. Treatment

A thyroid and cervical lymph node sonography was performed with no abnormalities observed. However, considering the possibility of a concomitant and occult papillary carcinoma in the thyroid, fully informed written consent had been obtained from the patient and total thyroidectomy (TT) with prophylactic bilateral central neck dissection (excision of levels VI and VII lymph nodes) has been performed, once it is the optimal surgical treatment for thyroid carcinoma. No dissected lymph node was macroscopically suggestive of metastasis, as observed intraoperatively.

Considering the American Thyroid Association risk stratification (intermediate risk) [7, 17], it would be necessary to perform RIA therapy. Additionally, the possibility of this tumor being a metastasis from a thyroid cancer supported the rationale of TT.

## 5. Outcome and Follow-Up

The postthyroidectomy histopathological report revealed a 0.4 cm nonencapsulated papillary thyroid microcarcinoma (mPTC), follicular variant. The neoplastic mass displayed follicular architecture and was composed of cells with ground glass nuclei, nuclear grooves, and pseudoinclusions. No extrathyroidal involvement and vascular or neural invasion were observed. There were no metastasis in the 14 lymph nodes resected, and the surgical margins were free. This absence reinforces the TDCCa diagnosis and makes less likely the differential diagnosis of an occult PTC undergoing conspicuously cystic transformation. The thyroid tumor was staged as pT1a pN0 Mx.

The patient had negative thyroglobulin and antithyroglobulin after surgery. Thyroid remnant ablation was achieved by the administration of 30 mCi of radioactive iodine (RAI-131) and posteriorly a whole-body scintigraphy showed zero uptake of the substance. It was followed by TSH suppression (0.1–0.5 mU/L) and the patient remains disease-free after 9 months of follow-up.

## 6. Discussion

There is no consensus on the optimal treatment of TDCCa mainly because of the lack of data from larger studies. Most authors agree that Sistrunk's procedure (SP), originally described in 1928, a block resection of the TDC along with the hyoid bone and the surrounding soft tissue towards the foramen cecum, is the first-choice surgery for TDCCa [6]. Patel et al. have shown that, in the presence of a clinically normal thyroid gland, the only factor that considerably affected outcome prognosis was the extent of surgery for the thyroglossal cyst itself. Simple cyst excision was inferior to SP (10-year survival rates being 95% and 75%, resp.), and total thyroidectomy was of no additional survival benefit [8].

Recently, total or subtotal thyroidectomy has been recommended if there is cyst wall invasion by the carcinoma or if the TDCCa is larger than 1.0 cm [7]. Although extension to surrounding soft tissue has been reported in 17% to 55% of all TDC malignancies [12, 18, 19], it is not known whether this has any prognostic impact [20]. In our case, it measured 1.4 cm and there was invasion of the capsule.

Prognostic risk group assessment was proposed to identify patients who would benefit from additional TT [7] and TT should be added only to high-risk patients [5]. However, Bakkar et al. reported a 62% rate of concomitant thyroglossal cyst and thyroid carcinomas. Similar high incidence of concomitant thyroid cancers, which may be occult in 25 to 56% of cases, results had been previously observed [20, 21].

Bakkar et al. also reported a 43% risk of missing thyroid malignancy in the setting of a sonographically normal thyroid gland and that the size of the thyroglossal cyst carcinoma could not serve as a predictive factor for the presence or absence of a concomitant thyroid carcinoma. Therefore, they concluded that selecting a subset of patients free of the risk of a concomitant thyroid cancer or free of the need for RAI ablation is a difficult task. Accordingly, they advocated the routine addition of TT to achieve comprehensive loco-regional control [18]. Other authors have this same rationale [19, 20].

Furthermore, TT permits systemic evaluation, treatment, and follow-up using serum markers [21]. Similarly, according to the risk stratification approach [7], a woman presenting a 1.4 cm tumor with cystic wall invasion would not be stated as low risk and would require additional TT.

Regional lymph node metastasis has been reported in up to 88% of TDCCa [22, 23]. This feature supported Hartl et al.'s statement that routine central compartment (level VI) dissection allows more precise lymph node staging, which may modify 131I ablation necessity [23]. It is important to notice that metastasis to the lateral compartment without central compartment involvement is not as rare as it is in thyroid gland cancers.

Despite this clear trend of lateral compartment nodes serving as primary stations for the spread of thyroglossal observed in some studies [12, 23], prophylactic lateral dissection in the absence of detectable nodal metastases has not been routinely recommended by any authors [5, 8, 24], even though regional neck dissection is recommended in high-risk group [7].

Therefore, the basis for a central neck dissection was to follow current guidelines for treatment of differentiated thyroid cancer, as recommended by Hartl et al. [23]. Furthermore, a lateral neck dissection can be performed secondarily if needed without an increase in surgical complications.

Prognosis of TDC papillary thyroid carcinoma seems to be similar to that of papillary carcinoma of the thyroid gland, as well as the long-term follow-up, although the reported follow-up time is short (median time of 12 y) and the number of patients is small. Mortality is low with only a few reported disease-related deaths [24, 25].

We have presented a case of a papillary thyroid carcinoma arising in a thyroglossal duct cyst, a rare condition with few cases published in the literature. There are many controversies about the tumor's origin and the extension of surgery needed, which makes the definition of many aspects related to its management and follow-up difficult. Some characteristics which point to higher recurrence rates can guide treatment. The selection of patients which are likely to have worst prognosis and, consequently, will need a more aggressive treatment is of great importance and an individualized approach is the best option to improve patient outcome.

## Additional Points

(i) Thyroglossal duct cyst carcinoma is an extremely rare condition and usually an incidental finding. (ii) Surgery is the mainstay management for thyroglossal cyst carcinoma. However, the optimal surgical strategy remains controversial.

## References

[1] Ellis P. D. M., Van Nostrand A. W. P. (1977). The applied anatomy of thyroglossal tract remnants. *Laryngoscope*.

[2] Allard R. H. B. (1982). The thyroglossal cyst. *Head and Neck Surgery*.

[3] Boswell W. C., Zoller M., Williams J. S., Lord S. A., Check W. (1994). Thyroglossal duct carcinoma. *American Surgeon*.

[4] Brentano H. (1911). Struma aberrata lingual mit druzen metastasen. *Deutsche Medizinische Wochenschrift*.

[5] Ramírez Plaza C. P., López M. E. D., Carrasco C. E.-G., Meseguer L. M., Perucho A. D. L. F. (2006). Management of well-differentiated thyroglossal remnant thyroid carcinoma: time to close the debate? Report of five new cases and proposal of a definitive algorithm for treatment. *Annals of Surgical Oncology*.

[6] Sistrunk W. E. (1928). Technique of removal of cyst and sinuses of the thyreoglossal duct. *Surgery, Gynecology & Obstetrics*.

[7] Tharmabala M., Kanthan R. (2013). Incidental thyroid papillary carcinoma in a thyroglossal duct cyst—management dilemmas. *International Journal of Surgery Case Reports*.

[8] Patel S. G., Escrig M., Shaha A. R., Singh B., Shah J. P. (2002). Management of well-differentiated thyroid carcinoma presenting within a thyroglossal duct cyst. *Journal of Surgical Oncology*.

[9] Baglam T., Binnetoglu A., Yumusakhuylu A. C., Demir B., Askan G., Sari M. (2015). Does papillary carcinoma of thyroglossal duct cyst develop de novo?. *Case Reports in Otolaryngology*.

[10] Gupta N., Dass A., Bhutani M., Singhal S. K., Verma H., Punia R. P. S. (2014). Papillary carcinoma in thyroglossal duct cyst: an unusual case. *Egyptian Journal of Ear, Nose, Throat and Allied Sciences*.

[11] Hilger A. W., Thompson S. D., Smallman L. A., Watkinson J. C. (1995). Papillary carcinoma arising in a thyroglossal duct cyst: a case report and literature review. *The Journal of Laryngology & Otology*.

[12] Pellegriti G., Lumera G., Malandrino P. (2013). Thyroid cancer in thyroglossal duct cysts requires a specific approach due to its unpredictable extension. *The Journal of Clinical Endocrinology and Metabolism*.

[13] Gebbia V., Di Gregorio C., Attard M. (2008). Thyroglossal duct cyst carcinoma with concurrent thyroid carcinoma: a case report. *Journal of Medical Case Reports*.

[14] Kutuya N., Kurosaki Y. (2008). Sonographic assessment of thyroglossal duct cysts in children. *Journal of Ultrasound in Medicine*.

[15] Yang Y. J., Haghir S., Wanamaker J. R., Powers C. N. (2000). Diagnosis of papillary carcinoma in a thyroglossal duct cyst by fine-needle aspiration biopsy. *Archives of Pathology and Laboratory Medicine*.

[16] Edge S. B., Byrd D. R., Compton C. C. (2010). *AJCC Cancer Staging Manual*.

[17] Haugen B. R., Alexander E. K., Bible K. C. (2016). 2015 american thyroid association management guidelines for adult patients with thyroid nodules and differentiated thyroid cancer: the american thyroid association guidelines task force on thyroid nodules and differentiated thyroid cancer. *Thyroid*.

[18] Bakkar S., Biricotti M., Stefanini G., Ambrosini C. E., Materazzi G., Miccoli P. (2016). The extent of surgery in thyroglossal cyst carcinoma. *Langenbeck's Archives of Surgery*.

[19] Miccoli P., Minuto M. N., Galleri D., Puccini M., Berti P. (2004). Extent of surgery in thyroglossal duct carcinoma: reflections on a series of eighteen cases. *Thyroid*.

[20] Basu S., Shet T., Borges A. M. (2009). Outcome of primary papillary carcinoma of thyroglossal duct cyst with local infiltration to soft tissues and uninvolved thyroid. *Indian Journal of Cancer*.

[21] Manipadam J. M., Manipadam M. T., Thomas E. M. (2011). Thyroglossal duct carcinoma: a case series and approach to management. *World Journal of Endocrine Surgery*.

[22] Dzodic R., Markovic I., Stanojevic B. (2012). Surgical management of primary thyroid carcinoma arising in thyroglossal duct cyst: an experience of a single institution in Serbia. *Endocrine Journal*.

[23] Hartl D. M., Ghuzlan A. A., Chami L., Leboulleux S., Schlumberger M., Travagli J.-P. (2009). High rate of multifocality and occult lymph node metastases in papillary thyroid carcinoma arising in thyroglossal duct cysts. *Annals of Surgical Oncology*.

[24] Kermani W., Belcadhi M., Abdelkéfi M., Bouzouita K. (2008). Papillary carcinoma arising in a thyroglossal duct cyst: case report and discussion of management modalities. *European Archives of Oto-Rhino-Laryngology*.

[25] Vassilatou E., Proikas K., Margari N., Papadimitriou N., Hadjidakis D., Dimitriadis G. (2014). An adolescent with a rare midline neck tumor: thyroid carcinoma in a thyroglossal duct cyst. *Journal of Pediatric Hematology/Oncology*.

